# Mr. Hemingway on Puerperal Convulsions

**Published:** 1813-07

**Authors:** H. Hemingway

**Affiliations:** Earl's Heaton, near Leeds


*To the Editors of the Medical and Physical Journal.*

GENTLEMEN,

I BEG leave to present for insertion in your Journal the following case of Convulsions during Labor, which, by a prompt and decisive mode of treatment, terminated very satisfactorily.

April 21st, I was requested to attend the wife of W. B. of C——, near this place, who had been in labor several hours. On examination, I found the os uteri very little dilated and rigid, the membranes not ruptured, and the presentation natural. The woman appeared nearly desponding, on account of the circumstances attending a former labor, she having been delivered with the forceps. I represented the state of the labor to her, and left her, promising to see her again in a few hours. On my return, I found that she had been attacked with convulsions, about an hour before my arrival: her extremities were contracted and stiff, pulse nearly gone, breathing scarcely perceptible, and a peculiar wild appearance in her countenance. I immediately procured a strong mixture of brandy and water, and, with some difficulty, succeeded in getting her to swallow two tea cupfuls: this gave her the most decided relief; she became rational, strong pains followed, and the child was expelled in less than an hour afterwards, in a natural manner. My patient has recovered very well.

I am, Gentlemen,

Your obedient Servant,

*Earl's Heaton, near Leeds,* H. HEMINGWAY.

*May* 27, 1813.

